# At-home cancer screening: a solution for China and other developing countries with a large population and limited number of healthcare practitioners

**DOI:** 10.1186/s40880-017-0235-2

**Published:** 2017-08-21

**Authors:** Chao-Nan Qian

**Affiliations:** Department of Nasopharyngeal Carcinoma, State Key Laboratory of Oncology in South China, Collaborative Innovation Center for Cancer Medicine, Sun Yat-sen University Cancer Center, Guangzhou, 510060 Guangdong P. R. China

**Keywords:** The spectrum of cancer types, At-home cancer screening, Early detection rate, Standardized treatment, Lung cancer, Cervical cancer, Gastric cancer, Colorectal cancer, Breast cancer, HPV, Stool DNA

## Abstract

Five-year survival rate for patients with all cancers combined, in China, is only 30.9%, which is much lower than those in developed countries. The three main reasons for the low cancer curative rates in China include differences in the spectrum of cancer types, in early detection rates, and in the percentage of cancer patients receiving standardized treatment between China and developed countries. The most important mechanism for improving the curative rate is to improve early detection rates of major cancers in China using novel and affordable technologies that can be operated at home by the patients themselves. This attempt could be helpful in setting up a practical example for other developing countries with limited medical resources and a limited number of healthcare practitioners.

## Text

In China, the age-standardized 5-year survival rate for patients with all cancers combined is only 30.9% [1], which is much lower than that in the United States (68%) [2] and that in the United Kingdom (54.3%) [3]. There are three main reasons for the huge discrepancies of cancer treatment outcomes between China and developed countries: (1) differences in the spectrum of cancer types; (2) differences in early detection rates; and (3) differences in the percentage of cancer patients receiving standardized treatment.

## Spectrum of cancer types

In the US and other western countries, gastric, liver, and esophageal cancers are less common, whereas in China, these malignancies are consistently among the top 10 most common cancer types [4–6]. Patients with these three types of cancer usually have shorter survival in contrast to patients with prostate and breast cancers, the latter two being more common in the US and other western countries [7]. The differences in the spectrum of cancer types can hardly be changed within a decade. However, along with gradual changes in the Chinese lifestyle, as well as the application of vaccinations against hepatitis B and C virus infections, the incidence of gastric, esophageal, and liver cancers are expected to decline in the next two decades.

## Early detection rates and at-home cancer screening

Patient survival for many malignancies is mainly dependent on the stage of the disease. The most important reason underlying the discrepancies of the curative rates between China and developed countries is that early cancer detection rates are pretty low for most of the cancer types in China. For operable gastric cancers in China, stage I diseases only account for 18%, and approximately 59% are stage III–IV diseases [8]. Contrastingly, in Japan, early detection rates are very high, with over 60% of stage I diseases and 24% of stage III–IV diseases [9]. For breast cancer, stage I diseases account for only 13.5% of all cases in China, in contrast, it is 50.5% in the US [10]. For colorectal cancer, early detection rates in large city hospitals in China is 30% [11], whereas the average in the US is 38.5% [12]. Clearly, more endeavors need to be made to significantly increase the early detection rates of major cancers in China.

The application of cancer screening for major cancers in healthy populations using current medical technologies is impeded by two main difficulties. The first difficulty is the expensive cost of current technologies. Due to limited financial resources, the state-owned medical insurance agencies for most Chinese people cannot cover the annual workup for major cancers using traditional technologies, e.g., chest X-ray for lung cancer, let alone using more accurate yet expensive technologies, e.g., low-dose computed tomography scanning for lung cancer. Another difficulty is the limited number of healthcare practitioners in China to complete population-based screening for major cancers using traditional technologies, which are highly dependent on professionals for operation [8].

Some novel and inexpensive technologies need to be invented to simultaneously overcome these two difficulties in China and other developing countries facing similar challenges. Encouragingly, several promising techniques for at-home cancer screening have been approved for clinical practice in the US. By detecting tumor specific *KRAS* mutations, abnormal *NDRG4*, and bone morphogenetic protein 3 (*BMP3*) methylation, plus hemoglobin immunoassays of stool samples, a company named *Exact Sciences* has developed an at-home screening kit for detecting colorectal cancer, as well as advanced precancerous lesions, with satisfying sensitivity and specificity [13]. For cervical cancer screening, technologies based on self-collected vaginal samples for human papillomavirus infection (HPV) testing are available and currently under clinical validation [14, 15]. More promisingly, highly sensitive nanoarray sensors for exhaled volatile organic compounds have recently been developed for early detection of lung cancer using breath samples, which can even detect epidermal growth factor receptor (*EGFR*) mutation for differential diagnosis [16, 17].

Before widespread use of at-home screening technologies in China, validation trials need to be conducted to optimize the techniques and to customize the analytical criteria for the Chinese population. These endeavors could be made by establishing several institutions in different parts of the country responsible for conducting clinical trials, optimizing screening systems, and routinely testing and storing the samples.

## Standardized treatment

It is estimated that over 60% of cancer patients in China are not treated in specialized cancer hospitals or cancer centers but treated in general hospitals. Radiotherapy in some general hospitals is limited and/or inaccessible, therefore, a significant portion of cancer patients do not receive standard treatment following international guidelines. Another challenge is the shortage of oncological specialists in China. China has launched nationwide residency training programs in 2014, including a radiation oncologist residency training program. However, the long time-discussed specialist training programs for medical oncologists and surgical oncologists have not yet been launched. It is expected that by improving the education and training systems for oncological specialists in China, more and more patients will be treated in accordance with international guidelines.

In conclusion, many efforts need to be made to improve the survival rates of cancer patients in China. The most important one is to improve early detection rates of major cancers in China using novel and affordable technologies allowing at-home sample collection by the patients themselves. All of these efforts could be helpful in setting up a successful example for other developing countries with limited medical resources and a limited number of healthcare practitioners.
